# Correction: Sensory cilia: How molecular motors can shape ciliary tip organization and diversity

**DOI:** 10.1083/jcb.20260302704272026c

**Published:** 2026-05-18

**Authors:** Bénédicte Durand

Vol. 225, No. 5 | https://doi.org/10.1083/jcb.202603027 | April 22, 2026

After publication of the article, the author discovered that they had failed to cite the first study to support the statement that “different kinesin motors moving along the cilium shape the ciliary endings of its sensory neurons.” They have now added this citation to the text and reference list.

Evans, J.E., J.J. Snow, A.L. Gunnarson, G. Ou, H. Stahlberg, K.L. McDonald, and J.M. Scholey. 2006. Functional modulation of IFT kinesins extends the sensory repertoire of ciliated neurons in *Caenorhabditis elegans*. *J. Cell Biol.* 172:663–669. https://doi.org/10.1083/jcb.200509115.

The affected text, from the fifth paragraph of this Spotlight, is shown in bold below:

Among the possible molecular pathways that regulate ciliary diversity, the intraflagellar transport (IFT) is a privileged candidate. This pathway is involved in building almost all cilia. It is responsible for conveying cargoes required to assemble and maintain the cilium (Lacey and Pigino, 2025). This transport process involves kinesin and dynein motors, and disturbances to this process can result in the complete absence of ciliary assembly or more subtle signaling defects in milder conditions. Given its critical role in cilium assembly, it was anticipated that this transport machinery would also be involved in the formation of cilium-specific features. **This hypothesis was strongly supported by initial seminal work in *C. elegans*, showing that different kinesin motors moving along the cilium shape the ciliary endings of its sensory neurons (Evans et al., 2006; Mukhopadhyay et al., 2007)**. While heterotrimeric kinesin II drives the anterograde movement of IFT in all organisms studied so far, a second homodimeric kinesin, OSM-3/KIF17, has been identified as essential for building the distal segment, composed of singlet microtubules, in a specific subtype of sensory cilia in the worm. Similarly, in vertebrates, KIF17 has been shown to be necessary for forming the outer segment of photoreceptors (Insinna et al., 2008). Interestingly, it has been shown that tubulin glutamylation regulates these IFT motors in a cell type–specific manner to build cilium-specific shapes (O’Hagan et al., 2017). In addition, a balance in phosphorylation and dephosphorylation events of OSM-3 regulates its activity while being transported by kinesin II to the distal segment to fulfill its function (Huang et al., 2025). Taken together, these observations suggest that modulating IFT motors may be sufficient to sustain diverse organization of ciliary endings.
